# Long-term complications and outcomes of augmentation cystoplasty in children with neurogenic bladder

**DOI:** 10.1038/s41598-024-54431-z

**Published:** 2024-02-20

**Authors:** Jei-Wen Chang, Fang-Cheng Kuo, Tzu-Ching Lin, Tai-Wai Chin, Ling-Yu Yang, Hsin-Hung Chen, Yu-Hua Fan, Hui-Hsin Yang, Chin-Su Liu, Hsin-Lin Tsai

**Affiliations:** 1 Department of Pediatrics, Taipei Veterans General Hospital, Taipei, Taiwan; 2 Institute of Emergency and Critical Care Medicine, School of Medicine, National Yang Ming Chiao Tung University, Taipei, Taiwan; 3 Faculty of Medicine, School of Medicine, National Yang Ming Chiao Tung University, Taipei, Taiwan; 4 Division of Pediatric Surgery, Department of Surgery, Taipei Veterans General Hospital, No. 201, Sec. 2, Shih-Pai Road, Bei-Tou District, Taipei, Taiwan; 5 Department of Pediatric Surgery, Changhua Christian Children Hospital, Changhua, Taiwan; 6 Department of Medical Education, Taipei Veterans General Hospital, Taipei, Taiwan; 7 Department of Neurosurgery, Neurological Institute, Taipei Veterans General Hospital, Taipei, Taiwan; 8 Department of Urology, Taipei Veterans General Hospital, Taipei, Taiwan

**Keywords:** Augmentation cystoplasty, Complication, Neurogenic bladder, Pediatric, Nephrology, Urology

## Abstract

Augmentation cystoplasty (AC) is an effective surgical procedure for patients with neurogenic bladder whenever conservative treatments have failed. The present study aimed to determine the risks of metabolic complications, malignancy, long-term outcomes and histopathologic changes of native bladder and the augmented intestine after AC in children with neurogenic bladder. Pediatric patients < 18 years who underwent AC between 2000 and 2020 were enrolled. Early postoperative complications, long-term outcomes and histopathologic changes in mucosal biopsies of native bladder and the augmented intestine after AC were reviewed. Twenty-two patients with a mean age of 7.6 ± 4.4 years were included. The ileum was used in 19 patients and the sigmoid colon in 3 patients. The length of hospital stay was 14.8 ± 6.8 days. Post-operatively, the urinary continence rate improved from 22.7 to 81.8% (*p* < 0.001). Hydronephrosis resolved in 17 of 19 patients. Vesicoureteral reflux resolved in 16 (64.0%) of the refluxing ureter units and was downgraded in 7 (28.0%). Grades of hydronephrosis and reflux significantly improved following AC (*p* < 0.001). The estimated glomerular filtration rate also significantly increased (*p* = 0.012). Formation of urinary tract stones was the most frequent late complication (in 8 patients, 36.4%). Life-threatening spontaneous bladder perforation occurred in 1 patient. After a mean follow-up of 13.4 ± 5.9 years, there were no cases of mortality, new-onset symptomatic metabolic acidosis, or changes in serum electrolytes. Of the 17 patients who were followed for > 10 years, no cases of malignancy or metaplastic changes were identified in the native bladder or augmented bowel epithelium. AC is a safe and effective procedure with low surgical and metabolic complication rates. In addition, AC provides a satisfactory continence rate and long-term protection of renal function, increases functional capacity, and regresses reflux and hydronephrosis. Individualized surveillance is recommended for the early identification of urolithiasis and metabolic disturbances.

## Introduction

Augmentation cystoplasty (AC) is used as a last resort to reduce intravesical pressure, and it is indicated in children with reduced bladder compliance, low bladder capacity or refractory overactive bladder in whom medical treatment with anticholinergics, the β-3 adrenergic agonist mirabegron, and detrusor injections of botulinum toxin has failed^1^. Although great progress has been made in tissue engineering, the ideal material for use in AC has yet to be developed^2^. A variety of different segments of the bowel have been used for AC, although ileocystoplasty remains the most common type of AC.

Even though AC provides a functional reservoir to achieve urinary continence, protects the upper urinary tract and improves the quality of life, concerns remain about the high rate of long-term complications including urolithiasis, metabolic imbalance, bowel dysfunction and life-threatening bladder perforation^3–7^. Another major concern with AC is the potential increased risk of malignant transformation in the native and augmented bladder or at the vesicointestinal junction^8^. The risk of developing malignancy has been reported to be around 7–8-fold and 14–15-fold higher for patients augmented with ileum or colon and stomach, respectively^9^. Most cases of malignancy are diagnosed at an advanced stage. However, data related to independent risk factors, the timing of routine surveillance with urine cytology, cystoscopy and biopsies for the early detection of urologic malignancy remain controversial^10–12^.

Recent studies have reported a decrease in the use of AC^13,14^. While the exact cause for this is unknown, potential reasons may be related to concerns regarding the long-term risks of life-threatening complications, metabolic abnormalities, malignancy and mortality following AC. Most previous studies have only reported the short-term outcomes of AC, and controversy still exists regarding complications. To the best of our knowledge, no pediatric series has explored the long-term outcomes and histopathologic changes in cystoscopic mucosal biopsies of native bladder and the augmented intestine after AC. Therefore, we conducted this study to determine the risks of metabolic complications and malignancy and long-term functional outcomes in children who underwent AC with ileum and colon at a tertiary center.

## Results

### Patient characteristics and operative variables

AC was performed in 22 pediatric patients at a mean age of 7.6 ± 4.4 years, of whom 6 were boys and 16 were girls. The mean pre-operative body mass index (BMI) was 17.3 ± 3.2 kg/m^2^. Overall, 8 (36.4%) of the children were overweight/obese. The etiologies of neurogenic bladder included a history of myelomeningocele (n = 19), anorectal malformation with vertebral anomalies (n = 1), a history of transverse myelitis (n = 1), and posterior urethral valves (n = 1). Among the 22 patients, 19 (86.4%) were ambulatory with or without supportive devices such as ankle foot/knee ankle foot orthoses, while 3 (13.6%) were wheelchair dependent. The ileum was used in 19 patients and sigmoid colon in 3 patients. The mean lengths of the ileum and colon used for cystoplasty were 20.9 ± 3.5 and 18.3 ± 2.9 cm, respectively. The ileum segment was harvested a mean 20.9 ± 4.7 cm from the ileocecal valve. The mean follow-up period was 13.4 ± 5.9 years with at least 10 years, 5–10 years and 2–5 years in 17 (77.3%), 2 (9.1%) and 3 (13.6%) patients, respectively. The mean patient age at the end of this study was 20.9 ± 6.9 years.

### Early postoperative complications

The early postoperative complications are shown in Table 1. No intraoperative complications occurred during bladder augmentation. The mean operative times were 236.6 ± 65.4 min. The mean estimated blood loss was 79.7 ± 78.9 mL. Seven patients (31.8%) developed a total of 8 early postoperative complications, including urinary tract infections (UTIs) in 3 (13.6%), acute kidney injury (AKI) in 2 (9.1%), prolonged postoperative ileus in 1 (4.5%), bowel-bladder anastomotic leak in 1 (4.5%), and bowel obstruction in 1 (4.5%) patient which was treated with exploratory laparotomy. One AKI was caused by postoperative vesicoureteral anastomotic swelling which recovered after insertion of a 5-Fr nasogastric tube to the bilateral ureter. The other AKI was caused by urinary sepsis. After treatment, both patients with AKI recovered fully. In addition, urine leakage spontaneously resolved after Foley insertion. The mean duration of postoperative hospital stay was 14.8 ± 6.8 days. The overweight/obese patients did not have a significantly increased risk of overall early postoperative complications (12.5% vs 42.9%, *p* = 0.193), blood loss (70.0 ± 78.6 vs 85.2 ± 81.5 mL, *p* = 0.705), operative time (246.9 ± 57.1 vs 230.7 ± 71.0 min, *p* = 0.373), or hospital stay (12.3 ± 5.0 vs 16.3 ± 7.5 days,* p* = 0.170).

**Table 1:** Early and long-term complications after augmentation cystoplasty.

Variable	n (%)
Early complication	
Postoperative UTI	3 (13.6%)
AKI	2 (9.1%)
Bowel-bladder anastomotic leak	1 (4.5%)
Ileus	1 (4.5%)
Small bowel obstruction	1 (4.5%)
Late complication	
Metabolic complications	
New-onset hyperchloremic metabolic acidosis	0 (0.0%)
Electrolyte disturbances	0 (0.0%)
Urinary stone	
Kidney	3 (13.6%)
Urinary bladder	5 (22.7%)
Bowel disturbance	0 (0.0%)
Small bowel obstruction	0 (0.0%)
Intra-abdominal fluid collection	1 (4.5%)
Perforation	1 (4.5%)
Malignancy	0 (0.0%)

*AKI* acute kidney injury, *UTI* urinary tract infection.

### Medical complications and perforation

The late postoperative complications are shown in Table 1. The mean last postoperative blood pH value was 7.35 ± 0.05, and the mean bicarbonate and serum total CO_2_ values were 24.5 ± 2.1 mmol/L and 22.8 ± 2.4 mmol/L, respectively. Two patients had low bicarbonate levels caused by a deterioration in renal function requiring oral sodium bicarbonate supplements. No new-onset postoperative hyperchloremic metabolic acidosis was noted at the last follow-up. None of the patients were initiated on prophylactic vitamin B12 supplementation after surgery. During regular follow-up, only 1 patient had vitamin B12 deficiency. The mean vitamin B12 levels were 448.8 ± 168.1 and 575.7 ± 274.4 pg/mL in the patients who received ileal and colon bladder augmentation, respectively, and the difference was not significant. The hemoglobin and mean corpuscular volume were 13.2 ± 1.7 g/dL and 86.9 ± 7.5 fL, respectively. None of the patients had pernicious anemia, and all of the patients had normal serum sodium, potassium, chloride, calcium and phosphorus levels. None of the patients had gastrointestinal symptoms such as small bowel obstruction, chronic diarrhea, malabsorption syndrome or gallstone formation. During a total of 294.2 patient-years, 1 patient had a spontaneous perforation at an augmented bowel segment with overwhelming *Escherichia coli* (*E. coli*) sepsis at 3.4 years following AC. She was successfully managed with exploratory laparotomy, drain placement, maximal bladder drainage, closure of the perforation site, and appropriate antibiotic therapy.

### Asymptomatic bacteriuria and antimicrobial susceptibility patterns

All of the 22 (100%) patients had asymptomatic bacteriuria at last follow-up. The most common organism isolated was *E. coli* (n = 18), followed by *Klebsiella pneumonia* (n = 1)*, Citrobacter koseri* (n = 1), *Streptococcus agalactiae* (n = 1), and *Morganella morganii* (n = 1). Seventeen of the *E. coli* strains were sensitive to cefazolin (94.4%), and 10 (55.6%), 7 (38.9%) and 4 (22.2%) *E. coli* isolates showed resistance to ciprofloxacin, amoxicillin and trimethoprim/sulfamethoxazole, respectively. No extended-spectrum β-lactamase producing *E. coli* were identified. One *E. coli* (5.6%) strain was resistant to ≥ 3 classes of antimicrobials (multidrug resistant). Isolated *Klebsiella pneumonia, Citrobacter koseri, Streptococcus agalactiae* and *Morganella morganii* were sensitive to all common antibiotics tested.

### Stone formation

Calculi occurred in 8 patients after augmentation (7 with ileum segment and 1 with a colon segment) including bladder calculi in 5 patients at 5.3 ± 5.2 years post AC, and renal calculi in 3 patients at 8.1 ± 5.3 years post AC (Table 1). Among the patients with bladder stones, 1 and 1 patient presented with microscopic and gross hematuria, respectively. One patient with renal stones presented with loin pain. The other patients were asymptomatic and diagnosed during routine follow-up. Bladder stone recurrence occurred in 3 patients. All of the bladder stones were successfully managed with endoscopic procedures. One renal stone required extracorporeal shock wave lithotripsy, and 2 patients with small renal stones were treated with conservative management. Only one vesicle stone was analyzed, which showed calcium oxalate monohydrate. Stone formation was similar in the patients who underwent ileocystoplasty and sigmoidocystoplasty (36.8% vs 33.3%, *p* = 1.000).

### Upper urinary tract dilatation (UTD)

The status of preoperative and postoperative hydronephrosis is shown in Table 2. Preoperatively, 19 (86.4%) patients had hydronephrosis, of whom 4, 6 and 9 patients were with UTD P1, UTD P2 and UTD P3, respectively. Postoperatively, UTD P1 and UTD P3 were noted in 1 and 2 patients, respectively. The Wilcoxon signed-rank test showed that the overall postoperative hydronephrosis grades improved significantly (*p* < 0.001).

**Table 2:** Renal and functional outcomes after augmentation cystoplasty.

Variable	Preoperatively	Postoperatively	*p* value
Hydronephrosis			< 0.001
No hydronephrosis	3 (13.6%)	19 (86.4%)	
UTD P1	4 (18.2%)	1 (4.5%)	
UTD P2	6 (27.3%)	0 (0.0%)	
UTD P3	9 (40.9%)	2 (9.1%)	
Number of renal units with VUR (n = 44)			< 0.001
No VUR	19 (43.2%)	35 (79.5%)	
Grade I	4 (9.1%)	4 (9.1%)	
Grade II	0 (0.0%)	2 (4.5%)	
Grade III	4 (9.1%)	1 (2.3%)	
Grade IV	9 (20.5%)	2 (4.5%)	
Grade V	8 (18.2%)	0 (0.0%)	
Renal function outcome			
Creatinine (mg/dL)	0.8 ± 0.6	0.9 ± 0.8	0.230
eGFR (mL/min/1.73 m^2^)	80.6 ± 33.9	98.3 ± 39.5	0.012
CKD stage			0.115
CKD stage 1	8 (36.4%)	13 (59.1%)	
CKD stage 2	8 (36.4%)	6 (27.3%)	
CKD stage 3	5 (22.7%)	2 (9.1%)	
CKD stage 4	1 (4.5%)	0 (0.0%)	
CKD stage 5	0 (0.0%)	1 (4.5%)	
Urinary continence	5 (22.7%)	18 (81.8%)	< 0.001

*CKD* chronic kidney disease, *eGFR* estimated glomerular filtration rate, *UTD* urinary tract dilation, *VUR* vesicoureteral reflux.

### Vesicoureteral reflux (VUR)

As shown in Table 2, preoperatively, 18 patients had VUR, including 7 with bilateral and 11 with unilateral reflux. There were a total of 25 refluxing ureter units, including Grade I (n = 4), Grade III (n = 4), Grade IV (n = 9), and Grade V (n = 8) reflux. Five patients had received prior anti-reflux treatment before AC. Six refluxing ureters had been reimplanted prior to bladder augmentation in 4 patients, and 1 patient received bilateral subureteric injections of dextranomer/hyaluronic acid copolymer (Deflux, Q-Med, Uppsala, Sweden). Furthermore, simultaneous augmentation and ureteral reimplantation was performed in 6 patients (9 ureters). In the remaining patients, reflux was not corrected surgically. Following the augmentation procedure, resolution of VUR occurred in 16 (64.0%) of the 25 refluxing ureter units, and VUR was downgraded in the other 7 (28.0%) units. No change in reflux was noted in 2 (8.0%) of the refluxing ureter units. The VUR grades were significantly lower after surgery compared with the preoperatively (p < 0.001, Table 2).

### Chronic kidney disease (CKD) stage

Data of CKD are shown in Table 2. Preoperatively, unilateral renal atrophy was found in 7 patients. The preoperative renal function was normal (estimated glomerular filtration rate [eGFR] > 90 mL/min/1.73 m^2^) in 8 of the 22 patients (36.4%), 8 patients (36.4%) had CKD stage 2, 5 patients (22.7%) had CKD stage 3, and 1 (4.5%) patient had CKD stage 4. The preoperative serum creatinine level and eGFR were 0.8 ± 0.6 mg/dL and 80.6 ± 33.9 mL/min/1.73 m^2^, respectively. Four patients had AKI preoperatively, which improved significantly postoperatively. At last follow-up, the serum creatinine level, eGFR and the cystatin C level were 0.9 ± 0.8 mg/dL, 98.3 ± 39.5 mL/min/1.73 m^2^ and 1.0 ± 0.5 mg/L, respectively. The eGFR at last follow-up was significantly higher than that preoperatively (*p* = 0.012, Table 2). During the follow-up period, the CKD stage remained stable or improved in 9 and 10 patients, respectively. The other 3 patients had a slow progression of CKD after AC despite improvements in VUR or hydronephrosis. Of these 3 patients, 1 progressed from CKD stage 3 to stage 5 with an eGFR of 13.5 mL/min/1.73 m^2^, and the other 2 patients progressed from CKD stage 1 to stage 2, with eGFRs of 83.1 and 85.0 mL/min/1.73 m^2^, respectively Although most patients remained with stable or better CKD stage, no statistical differences were observed in serum creatinine and CKD stage following AC (Table 2). Elevated serum creatinine levels (> 1.2 mg/dL and > 0.9 mg/dL in males and females, respectively) and β2-microglobulin levels (> 2295 ng/mL) were detected in 5 (22.7%) and 5 (22.7%) patients, respectively, but elevated cystatin C levels (> 0.95 mg/L) were detected in 8 (36.4%) patients.

### Urinary continence

Preoperatively, 16 (72.7%) patients were managed with clean intermittent catheterization (CIC) and 17 (77.3%) patients were diagnosed with varying degrees of urinary incontinence. Postoperatively, all patients continued or started to empty their bladder by CIC. No additional procedures such as sling or bladder neck reconstruction procedures were required in any of the patients. Eighteen (81.8%) of the 22 patients reported being completely dry. In addition, 3 (13.6%) patients had self-reported stress incontinence with filling detrusor pressure < 40 cmH_2_O, while 1 (4.5%) patient had overflow incontinence affected his social activities. Among the patients with stress incontinence, one showed low-normal compliance and an open bladder neck in postoperative video-urodynamic studies. The patient with urinary incontinence secondary to overflow incontinence was confirmed on a video-urodynamic examination, which showed an open bladder neck, low bladder compliance and an end filling intravesical pressure > 40 cmH_2_O. AC significantly improved the urinary continence rate (81.8% vs 22.7%, *p* < 0.001, Table 2).

### Bladder capacity and urodynamic studies

Preoperatively, the urodynamic diagnosis was low bladder capacity in 14 (63.6%) patients, reduced compliance in 17 (77.3%), detrusor areflexia in 6 (27.3%), and detrusor hyperreflexia in 10 (45.5%). The preoperative cystometric bladder capacity was 105.9 ± 65.7 mL, and the average cystometric bladder capacity was 53.2 ± 27.4% of the expected bladder capacity (EBC). Postoperatively, 14 patients completed urodynamic studies. The results of the last urodynamic study performed at 11.7 ± 5.9 years after surgery revealed that the urinary compliance was 29.1 ± 16.7 mL/cmH_2_O. The maximum cystometric bladder capacity was 426.9 ± 109.6 mL, and the ratio of maximum cystometric bladder capacity/EBC was 110.6 ± 22.4%. No patient had bladder capacity < 65% of EBC. For those who did not undergo urodynamic studies after AC, the bladder capacities measured by CIC were adequate for age. In addition, 1 patient had low compliance and 6 patients had low-normal compliance. One patient with bilateral grade I VUR had a detrusor leak-point pressure (DLPP) > 40 cmH_2_O. Table 3 shows the association between the preoperative and postoperative urodynamic parameters with CKD stage. The preoperative cystometric bladder capacity was higher (128.4 ± 79.2 vs 94.6 ± 57.8 mL, *p* = 0.314) and cystometric bladder capacity/EBC was lower (50.8 ± 20.7 vs 54.4 ± 30.8%, *p* = 0.941) in the patients with CKD stage 1 than in those with CKD stages 2–5, although the differences did not reach statistical significance. Higher maximum cystometric bladder capacity (444.4 ± 107.6 vs 368.7 ± 115.5 mL, *p* = 0.237), maximum cystometric bladder capacity/EBC (111.9 ± 25.6 vs 106.3 ± 5.2%, *p* = 0.612) and compliance (30.3 ± 13.7 vs 26.1 ± 25.3 mL/cmH_2_O, *p* = 0.322) were noted in the patients with CKD stage 1 than in those with CKD stages 2–5, although the differences did not reach statistical significance.

**Table 3:** Association between preoperative and postoperative urodynamic parameters with CKD stage.

Variable	CKD stage 1	CKD stages 2–5	*p* value
Preoperatively			
Cystometric bladder capacity (mL)	128.4 ± 79.2	94.6 ± 57.8	0.314
Cystometric bladder capacity/EBC (%)	50.8 ± 20.7	54.4 ± 30.8	0.941
Postoperatively			
Cystometric bladder capacity (mL)	444.4 ± 107.6	368.7 ± 115.5	0.237
Cystometric bladder capacity/EBC (%)	111.9 ± 25.6	106.3 ± 5.2	0.612
Compliance (mL/cmH_2_O)	30.3 ± 13.7	26.1 ± 25.3	0.322

*CKD* chronic kidney disease, *EBC* expected bladder capacity.

### Cystoscopy and microscopic examinations of the native bladder and augmented intestine

All patients also had negative urine cytology results. Among the 17 patients who received cystoscopy, no suspicious macroscopic lesions were found in any patient except for mucus production from enteric segments. The intestinal mucosa could be easily differentiated from the native bladder by its color and villous appearance. Dysplasia was noted in 1 (5.9%) native bladder biopsy, while chronic inflammatory changes were noted in 16 (94.1%) and 17 (100%) of native bladder and intestinal biopsies, respectively. No cases of malignancy or metaplastic changes were identified in the native bladder biopsies or bowel epithelium.

## Discussion

The primary goal of treatment of neurogenic bladder is to protect the upper urinary tract from damage. Management of neurogenic bladder is a complex issue and requires an individualized care plan approach based on upon the results of urodynamic studies^15^. Currently, antimuscarinic agents including oxybutynin, tolterodine, solifenacin and darifenacin are used as first-line pharmacologic therapy. Evidence suggests that a combination of two or more antimuscarinic agents can provide effective, well-tolerated treatment in children. β-3 agonists such as mirabegron are newly approved alternative or adjunctive therapy to treat neurogenic detrusor overactivity in children^16^. Selective α-blockers appear to be effective for improving bladder emptying. In patients who have become refractory to medical treatment, botulinum toxin type A injection is a favorable intervention to postpone or avoid aggressive reconstructive surgery^17^.

AC plays an important role in the management of refractory neurogenic bladder dysfunction in children when medical management and minimally invasive therapy fail. However, it is associated with serious surgical early and late complications as well as an increased risk of malignancy. In this study, we found that AC had acceptable immediate postoperative morbidity without severe complications. Consistent with the results in previous studies, ileum was the most commonly used segment in this study. Owing to reabsorption of sodium, ammonium, and chloride from the incorporated bowel segment as well as an increased loss of potassium and bicarbonate into the urine, metabolic disturbances including hyperchloremic and hypokalemic metabolic acidosis have been widely reported^18,19^. In our series, two patients with preoperative metabolic acidosis still had metabolic acidosis postoperatively accompanied by decreased renal function. There were no cases of new-onset metabolic acidosis postoperatively, in contrast to other reported series^20^. Previous studies have reported adaptive changes of intestinal mucosa after AC such as villous atrophy^21^, which may explain the occurrence of subtle and asymptomatic metabolic acidosis. However, persistent acidosis may have a negative impact on bone mineral density and result in an increased risk of osteoporosis^22^ and cause growth retardation in children. Therefore, long-term monitoring is required to assess the metabolic consequences, especially in children with impaired renal function.

Not all patients, and in particular neuropathic children, can achieve complete emptying of the augmented bladder by spontaneous voiding after AC, and the reported overall rate of CIC for emptying ranges from 26 to 100%^4,23,24^. Asymptomatic bacteriuria is common in CIC patients. In this study, all of the patients with an augmented bladder had asymptomatic bacteriuria, and *E. coli* was the most common causative pathogen. The low overall resistance rate of extended-spectrum β-lactamase and multidrug resistant and the high quinolone resistance in *E. coli* in our isolates may be explained by the high levels of quinolone resistance in Taiwan^25^ rather than the overprescription of previous antibiotics for UTIs in our patients. Asymptomatic or symptomatic bacteriuria may be associated with the formation of bladder calculi, and stone formation following AC has been reported to occur in 10.5–52% of patients^26–28^. In our study, the prevalence of urolithiasis was 36.4%, which is consistent with previous studies. Risk factors for the development of stones include patients with an underlying diagnosis of bladder or cloacal exstrophy^28^, UTI^26^, bladder neck surgery^29^, using ileum^3,26,28^, presence of an abdominal stoma^28,30^, and an immobile patient with sensory impairment^30^. Prophylactic bladder irrigation can reduce the risk of bladder calculi^5^. There are currently no standard guidelines regarding the frequency, specific type of fluid, additive, or volume of irrigation^26,30,31^. The reported irrigants include tap water, saline, 20% urea^26^, *N*-acetylcysteine, and gentamicin^31^and studies performed irrigation with variable volumes of fluid, but usually 120–250 mL per irrigation^5,30,31^, on a daily basis^5,31^. However, strategies are still required to overcome poor adherence to bladder irrigation in the future^31^. As in the general population, fluid status has been proven to be an important factor for stone formation post AC^32,33^, and patients are advised to maintain proper hydration with an individualized CIC regimen according to postoperative augmented bladder capacity.

Spontaneous bladder perforation is an uncommon but life-threatening condition that requires prompt diagnosis and treatment. The reported incidence of bladder perforation ranges from 5 to 13%^28,34,35^. Some patients have been reported to develop re-perforation^36^. Use of the sigmoid colon for augmentation, bladder-neck surgery, high bladder pressure, noncompliant catheterization and abdominal trauma have been associated with an increased risk of perforation, whereas the presence of a continent cutaneous catheterizable channel has been associated with a decreased risk^6,34,37^. In the present study, the relatively low incidence of spontaneous bladder perforation may be explained by the low rate of additional procedures to achieve continence and strict adherence to CIC postoperatively. As with most previous studies, the cause of perforation appeared to be related to impaired sensation combined with bladder overdistension caused by delayed catheterization in our patient. Spontaneous perforation of an infected bladder in AC patients can cause secondary peritonitis and shock. Prompt intervention with emergency laparotomy to repair the bladder perforation and concurrent broad-spectrum antibiotic coverage based on previous urine cultures remain the mainstay of treatment in cases of sepsis.

The use of ileum for AC can result in malabsorption of vitamin B12 and bile acid^38,39^. Depending on the desired capacity, approximately 15–40 cm of ileum at least 15–20 cm proximal to the ileocecal valve is usually used for cystoplasty. One study reported that a high percentage (62%) of patients had low or low-normal serum vitamin B12 beginning at 7 years postoperatively, and that the risk increased with time^39^. In the present study, none of the patients in whom the ileum was harvested at least 15 cm from the ileocecal valve developed vitamin B12 deficiency, and the ileum was harvested 10 cm away from the ileocecal valve in the only patient with mild vitamin B12 deficiency. Unlike most other water-soluble vitamins, vitamin B12 is stored in substantial amounts in the liver. Hence, it takes years to develop anemia after malabsorption of vitamin B12 begins. Therefore, those at risk should have their vitamin B12 status checked regularly for a long time.

VUR with deterioration of renal function is one of the most common complications of low bladder compliance. Postoperatively, the capacity of the bladder and intravesical pressure usually improve, thereby resulting in downgrading or spontaneous resolution of most reflux, making an anti-reflux procedure unnecessary^40,41^. As a consequence, there is postoperative improvement in upper UTD which protects the upper urinary tracts from long-term damage. As expected, our data showed marked improvements in hydronephrosis and resolution or downgrade of VUR. At our institute, we do not routinely perform ureteral reimplantation with AC. Similar to Wang et al.^42^, 27.3% of the patients in our study received concomitant ureteral reimplantation with AC, and this may have had a beneficial impact on the rate of VUR resolution. Furthermore, renal function stabilized or improved in 86.4% of the patients. In this study, myelomeningocele was the most common cause of neurogenic bladder. Creatinine-based formulae for eGFR have been shown to potentially overestimate the GFR in patients with spina bifida due to low muscle mass^43^. Serum levels of cystatin C are a superior marker than serum creatinine for kidney function and not related to gender, age, protein intake, and muscle mass. Post AC, we also measured cystatin C and β2-microglobulin, and found that 3 patients had normal creatinine and β2-microglobulin values with elevated cystatin C levels, which suggests that serum cystatin C may be a better early marker of a slightly reduced GFR. Many studies have shown that compliance < 10 mL/cmH_2_O and DLPP > 40 cmH_2_O were predictors of upper urinary tract damage^44,45^. However, other studies have failed to show compliance as a risk factor for renal deterioration^45,46^, which is consistent with our findings. Furthermore, we did not find a significant association between postoperative cystometric capacity and maximum cystometric bladder capacity/EBC with renal function.

Urinary incontinence is not only a medical problem but also a psychological and social problem, creating embarrassment and negative self‐perception. AC offers satisfactory outcomes with regards to continence and improvement of quality of life^18,47^. All of our patients were on CIC post-AC, and the overall continence rate of 81.8% is consistent with those reported in previous studies (from 53 to 100%) in patients with neurogenic detrusor overactivity after cystoplasty alone or with an artificial urinary sphincter^23,48,49^. Urinary continence after AC is determined by the combined effect of different factors and is a challenge for the pediatric urologic surgeon^49,50^. Medel et al. reported that a closed bladder neck in preoperative video-urodynamic studies was a predictor of post-AC continence^49^. However, most of the patients in our study underwent traditional urodynamic examinations rather than video-urodynamic studies preoperatively. Therefore, the predictive value of the preoperative appearance of the bladder neck on post-AC continence was not assessed in this study. Postoperative video-urodynamic studies demonstrated an open bladder neck in two of the 4 patients with persistent urinary incontinence in present study. CIC plays an important role in the management of neurogenic bladder and is usually required following AC. A higher rate of poor adherence to CIC has been shown among patients with persistent incontinence^51,52^. As AC can achieve a high permanent urinary continence rate, this may further enforce adherence to CIC to prevent UTI and renal deterioration.

A potential complication after AC is an increased risk of bladder cancer, however this risk has been disputed in recent studies^53,54^. Most cancers are diagnosed at an advanced stage and consequently have a very poor prognosis, and this has prompted the use of routine cystoscopy screening postoperatively^8,55^. However, the mean reported latency period to a diagnosis of cancer is 19 years, with the majority being over 10 years after the initial surgery. The necessity of long-term surveillance by cystoscopy is still controversial. Metcalfe et al.^3^ and Kispal et al.^6^ recommended repeated routine cystoscopy with or without biopsy commencing from 4 to 10 years following AC to detect premalignant changes early. In addition, Husmann et al.^9,53^ recommended that patients who have recurrent symptomatic UTIs (> 4 episodes/year), chronic perineal or bladder pain, gross or persistent microhematuria, abnormalities on ultrasound such as hydronephrosis, thickening of the bladder wall or suspicious image that does not change appearance with changes in position or with repeated washings, and those receiving immunosuppressive therapy should receive additional endoscopic and cytologic evaluations for the occurrence of cancer. Chronic bacterial infections of the urinary bladder can result in the formation of N-nitrosamines from urinary urea, which are carcinogenic. After a mean 13.4 ± 5.9 years of follow-up, we did not identify any cases of malignancy in our series. Chronic inflammation of the mucosa was found in all of the native and bowel epithelium, which was probably due to the high frequency of bacterial colonization caused by CIC. Unlike a previous study^56^, metaplastic changes of the urothelial and intestine mucosa were not found.

The strengths of this study are its long-term follow-up and detailed analysis of renal function and histological changes of the native bladder and augmented intestine. However, there are several limitations due to its retrospective design, and as the sample size is small. First, the results of this study may not be generalizable to other populations as we only reported the outcomes of AC at a single tertiary center. Second, the retrospective nature of the study may have under-reported the episodes of febrile UTIs, as the patients may not always have been admitted to our institution for febrile UTIs. Therefore, our outcome investigation did not include febrile UTIs. Prospective studies should be conducted to clarify the outcomes and complications. Third, in our cohort, irrigation with saline was given only in cases of excessive amounts of mucous or mucus-induced catheter obstruction rather than regular daily bladder irrigation. The lack of daily irrigation may have increased the risk of bladder stone formation and chronic infection. Fourth, detailed records from a catheterization diary before and after surgery were not assessed, because long-term recording may increase patient burden and adherence is often poor. Therefore, we assessed adherence to the prescribed CIC regimen including the number of CICs per day and the estimated volume per CIC by asking the patient and caregiver instead of using a catheterization diary during consequent Spina Bifida Multidisciplinary follow-up visits. Fifth, of the 22 participants included, 14 underwent postoperative urodynamic studies. In clinical practice, urodynamic studies may be performed only when the patients are symptomatic with unresolved urinary incontinence or VUR after AC. Therefore, we only assessed associations between the urodynamic parameters with CKD among the patients who underwent postoperative invasive urodynamic studies. Furthermore, preoperative serum levels of cystatin C were not available.

In conclusion, AC is a highly reliable treatment option for refractory neurogenic bladder in children. AC successfully increases bladder capacity and compliance, preserves renal function, improves anatomical deterioration of the upper urinary tract and provides urinary continence. Urolithiasis is the most frequent complication. AC was associated with a low metabolic complication rate in long-term follow-up in this study, and no cases of malignancy were identified. To avoid complications, we suggest strict adherence to CIC and individualized surveillance after AC for the early detection and treatment of complications.

## Methods

### Study design and subjects

Between 2000 and 2020, children (aged < 18 years) who underwent AC for neurogenic bladder confirmed by urodynamic studies at our institution were enrolled. Patients who did not complete a minimum 2 years of follow-up, and those who were lost to follow-up were excluded. Patient demographics, underlying diagnosis, comprehensive surgical and clinical information, and early and long-term postoperative complications were recorded. The Institutional Review Board of Taipei Veterans General Hospital approved this study (No. 2023-02-008AC). The requirement of informed consent was waived by the ethics committee of Taipei Veterans General Hospital due to the retrospective and observational nature of the present study. This study also was conducted in accordance with the Declaration of Helsinki statement.

### Preoperative implementation of CIC, evaluation and education

Preoperatively, early CIC was implemented in patients with detrusor areflexia, detrusor hyperactivity and/or detrusor sphincter dyssynergia to complete bladder emptying and thus avoid the consequent risk of infection and renal injury. Preoperative assessments included kidney function, serum biochemistry, renal ultrasonography for upper UTD, voiding cystourethrogram (VCUG), and urodynamic evaluations including compliance and bladder capacity. Before surgery, patients who were not on CIC preoperatively were instructed to catheterize four to five times a day to ascertain their motivation and ability to perform CIC to avoid serious complications. All patients and caregivers received preoperative education about the warning signs for urinary tract stones, perforation, and carcinoma complications. Preoperative BMI was calculated as weight in kilograms divided by the square of height in meters (kg/m^2^). Weight status was stratified into underweight/normal (BMI < 85th percentile) and overweight/obese (BMI ≥ 85th percentile) according to standard age- and sex-specific BMI values.

### Indications of concomitant ureteral reimplantation

Concomitant ureteral reimplantation was routinely performed between 2000 and 2001. From 2002, only patients with high grade VUR at a low bladder pressure and after intraoperative cystoscopy evaluation of the native bladder who were considered suitable for reimplantation received simultaneous ureteral reimplantation and AC.

### Perioperative bowel preparation

All patients undergoing AC received preoperative antibiotic bowel preparation with one dose of intravenous weight‐based cefazolin, metronidazole and gentamicin given at the induction of anesthesia. For patients using sigmoid colon, a cleansing enema was used for mechanical bowel preparation.

### Postoperative monitoring and follow-up

Postoperative follow-up consisted of regular laboratory evaluations and neo-bladder and renal ultrasound to prevent complications. Neo-bladder and renal ultrasound follow-up screening were done every 6–12 months, or symptomatic UTIs and hematuria. Postoperative VCUG was reperformed to assess outcomes after AC and in patients with repeated febrile UTIs and progressive UTD during an ultrasound examination. Urodynamic re-evaluations were recommended especially for those with persistent severe hydronephrosis, high-grade VUR, or urinary incontinence. Bladder compliance was calculated as the change in bladder volume divided by the change in detrusor pressure during filling (ΔV/ΔPdet), and expressed in mL/cmH_2_O. Normal bladder compliance was defined as a value of > 30 mL/cmH_2_O, and the cutoff-value used for low compliance was < 10 mL/cmH_2_O. Compliance between 10 and 30 mL/cmH_2_O was considered low-normal. A DLPP > 40 cmH_2_O indicated the risk of upper tract deterioration. At the latest follow up after AC, all patients received comprehensive surveillance surveys consisting of serum biochemistry including blood urea nitrogen, creatinine, cystatin C, β2-microglobulin, electrolytes, calcium, phosphate, venous blood gas, vitamin B12, urinalysis, urine culture, renal-bladder ultrasound and VCUG. Metabolic acidosis was defined as a venous pH less than 7.35 and bicarbonate concentration less than 22 mmol/L. A serum vitamin B12 level of less than 200 pg/mL indicated B12 deficiency. Bacteriuria was defined as a bacterial count greater than 10^5^ CFU per mL in a catheterized urine specimen. The status of continence was based on reports from the patients or their parents and urodynamic tests.

### Bladder capacity

The age-adjusted EBC was calculated according to the formula: EBC (mL) = [age (years) + 1] × 30 for children under 12 years^57^, and 400 mL for those older than 12 years^58^. Preoperative bladder capacity was measured during urodynamic examinations. Low bladder capacity was defined as less than 65% of EBC. Postoperative bladder capacity was estimated by patient-reported maximum catheterization urine volume or measured by urodynamic examinations if indicated.

### Upper tract deterioration: hydronephrosis severity and VUR grade

According to the UTD system^59^, the severity of hydronephrosis was categorized into UTD P1 (low risk), UTD P2 (intermediate risk), and UTD P3 (high risk of postnatal uropathies) based on ultrasound images of anterior posterior renal pelvis diameter, calyceal dilation, parenchymal thickness and appearance of kidney, ureter and urinary bladder. VUR was graded according to the International Reflux Study Group classification. VUR in our study was considered to be resolved when the VCUG study became normal with no further reflux. VUR was defined as being downgraded when there was a reduction from high-grade to low-grade VUR.

### Renal function evaluation

Renal function was assessed according to serum creatinine levels and eGFR using the bedside Schwartz formula in children under 18 years of age^60^, and the Modification of Diet in Renal Disease formula for adults^61^. According to the National Kidney Foundation criteria, CKD is classified into 5 stages based on eGFR; stage 1: eGFR > 90 mL/min/1.73 m^2^, stage 2: eGFR 60–89 mL/min/1.73 m^2^, stage 3: eGFR 30–59 mL/min/1.73 m^2^, stage 4: eGFR 15–29 mL/min/1.73 m^2^, and stage 5: eGFR < 15 mL/min/1.73 m^2^ or requiring dialysis. Renal function deterioration was defined as progression to a more severe CKD stage.

### Surveillance protocol of malignancy

The screening protocol of bladder malignancy was based on the recommendations from previous studies by the presence of clinical red flags including recurrent UTIs, bladder or pelvic pain, urinary symptoms such as gross hematuria, or persistent microhematuria and regular bladder ultrasound. Rigid diagnostic cystoscopy (Olympus, Tokyo, Japan) under general anesthesia was performed at least 10 years after AC or upon the occurrence of warning signs. A biopsy specimen was obtained from the native bladder and another from the enteric segment at the anastomotic border through a cystoscope using semi-rigid biopsy forceps (Richard Wolf GmbH, Knittlingen, Germany). The mucosal biopsies were examined with routine hematoxylin and eosin staining and evaluated for inflammation, metaplasia dysplasia and malignancy by a pathologist. Urinary cytology to identify the presence of atypical, suspicious, or malignant cells was used in conjunction with cystoscopy for the early detection of malignancy.

### Statistical analysis

Continuous variables were presented as mean ± standard deviation and categorical variables were presented as counts and percentages. The Mann–Whitney U test was used to compare differences in continuous variables between groups. Categorical variables were compared using the Fisher’s exact test. The Wilcoxon signed-rank test was used to compare hydronephrosis grades, VUR grades, creatinine, eGFR,and CKD stages before and after AC. McNemar’s test was used to test the change in urine continence after AC. All statistical analyses were performed using SPSS version 21.0 (SPSS Inc., Chicago, IL, USA). All p values less than 0.05 were considered significant.

## Data Availability

Data are not publicly available due to ethical reasons. Further inquiries can be directed to the corresponding author.
